# Serum and synovial fluid cytokine profiling in hip osteoarthritis: distinct from knee osteoarthritis and correlated with pain

**DOI:** 10.1186/s12891-018-1955-4

**Published:** 2018-02-05

**Authors:** Guomin Ren, Ian Lutz, Pamela Railton, J. Preston Wiley, Jenelle McAllister, James Powell, Roman J. Krawetz

**Affiliations:** 1 McCaig Institute for Bone and Joint Health, Faculty of Medicine, University of Calgary, 3330 Hospital Drive NW, Calgary, Alberta T2N 4N1 Canada; 2 Department of Surgery, University of Calgary, Calgary, Alberta Canada; 3 Charles Sturt University, Sydney, Australia; 4 Sports Medicine Centre, Faculty of Kinesiology, University of Calgary, Calgary, Alberta Canada; 5 Department of Anatomy and Cell Biology, University of Calgary, Calgary, Alberta Canada

**Keywords:** Osteoarthritis, Inflammation, Pain, Hip

## Abstract

**Background:**

Inflammation is associated with the onset and progression of osteoarthritis in multiple joints. It is well known that mechanical properties differ between different joints, however, it remains unknown if the inflammatory process is similar/distinct in patients with hip vs. knee OA. Without complete understanding of the role of any specific cytokine in the inflammatory process, understanding the ‘profile’ of inflammation in a given patient population is an essential starting point. The aim of this study was to identify serum cytokine profiles in hip Osteoarthritis (OA), and investigate the association between cytokine concentrations and clinical measurements within this patient population and compare these findings to knee OA and healthy control cohorts.

**Methods:**

In total, 250 serum samples (100 knee OA, 50 hip OA and 100 control) and 37 synovial fluid samples (8 knee OA, 14 hip OA and 15 control) were analyzed using a multiplex ELISA based approach. Synovial biopsies were also obtained and examined for specific cytokines. Pain, physical function and activity within the hip OA cohort were examined using the HOOS, SF-36, HHS and UCLA outcome measures.

**Results:**

The three cohorts showed distinct serum cytokine profiles. EGF, FGF2, MCP3, MIP1α, and IL8 were differentially expressed between hip and knee OA cohorts; while FGF2, GRO, IL8, MCP1, and VEGF were differentially expressed between hip OA and control cohorts. Eotaxin, GRO, MCP1, MIP1β, VEGF were differentially expressed between knee OA and control cohorts. EGF, IL8, MCP1, MIP1β were differentially expressed in synovial fluid from a sub-set of patients from each cohort. Specifically within the hip OA cohort, IL-6, MDC and IP10 were associated with pain and were also found to be present in synovial fluid and synovial membrane (except IL-6) of patients with hip OA.

**Conclusion:**

OA may include different inflammatory subtypes according to affected joints and distinct inflammatory processes may drive OA in these joints. IL6, MDC and IP10 are associated with hip OA pain and these proteins may be able to provide additional information regarding pain in hip OA patients.

**Electronic supplementary material:**

The online version of this article (10.1186/s12891-018-1955-4) contains supplementary material, which is available to authorized users.

## Background

Approximately 1 in 8 individuals are afflicted with Osteoarthritis (OA) and although it is more common in older populations, it is becoming a serious health and economical concern in young, active individuals [1, 2]. Therefore, it is essential to further understand the factors involved in the onset and progression of the disease, so that more efficient diagnostics and treatments can be developed.

While much of the research focus in OA has been directed towards the knee, it is necessary to examine if the pathways and mechanisms in knee OA are conserved in other joints. For example, in the hip, recent literature have provided insight into mechanical causes of hip OA including impingement and dysplasia [3], while others have focused on potential genetic predispositions with or without mechanical risk factors [4]. This suggests that, as in knee OA, there could be potentially many avenues of hip OA onset that eventually result in patients with diverse etiology converging later in the disease trajectory. Our previous work in knee OA looking at systemic and local inflammatory profiles suggests that inflammatory profiles are distinct in patients with knee OA from those without OA [5] and therefore that this approach may be able to discriminate systemic inflammatory differences between patients with knee vs. hip OA.

More generally, there have been a number of studies and many biochemical markers that have been identified in knee OA [6–10]. Historically groups have focused on cartilage metabolism markers including which demonstrate changes in concentration and fragment species with the onset and progression of OA [11–14]. Inflammation is known to be present in OA and the changes in individual inflammatory markers have been correlated with both severity and progression and OA [15, 16]. Regardless of the type of biochemical marker examined however, knee OA and hip OA are almost always grouped together or only one joint is examined within a given study [17]. Most of the common OA biochemical markers (collagen fragments, cartilage oligomeric matrix protein (COMP), etc.) have been tested on both knee and hip cohorts, but only a very few studies looked at both joints and run comparisons [17]. The uncontrolled variances (such as different definition of OA, cohort characteristic, experimental method) across studies make the results almost incomparable. Among the studies that contain both cohorts, the comparisons of biochemical markers between knees and hips are largely ignored [18, 19]. It is well known that mechanical properties differ between joints (knee vs. hip) [20, 21], but as far as we are aware only two studies have directly compared between cohorts of hip and knee OA patients: the effectiveness of bone metabolism for OA prognostic [22] and the difference of association between COMP and OA symptoms [23]. However, neither of them compared the absolute concentration of biochemical markers between two phenotypes.

In addition to biochemical markers, it is also important to gain a better understanding of pain in OA. Pain is the leading disabling symptom of OA [24]. According to the American College of Rheumatology (ACR) criteria for OA, pain is a necessary condition for clinical diagnosis of OA, with the support by other sufficient conditions such as age, radiographic evidences or physical examinations. OA pain is hard to quantify from patient to patient based on a number of confounding factors [25] and its inconsistency with other symptoms such as radiographic findings is not yet well understood. Recent studies have greatly improved our understanding of pain at the molecular level [26], and new biochemical markers have also been reported to be correlated with severity of OA pain in the past decade (Additional file 1: Table S1). Cytokines play an important role in the pain signal pathway from lesions to higher brain processing centers [27]. While cytokines can change the sensitivity of peripheral receptors to nociceptive input by a variety of mechanisms in experimental animal models [28–30], very few human studies have reported correlations between cytokines and OA pain. The chemokine sub-family of cytokines is also of specific interest in patients with arthritis since these small molecules are capable of recruiting additional immune and/or stem/progenitor cells to sites of injury and/or increased inflammation.

With our incomplete understanding of the role of any specific cytokine in the inflammatory process, understanding the ‘profile’ of inflammation in a given patient population is an essential starting point. Therefore, the primary aim of this study was to examine system and local inflammatory profiles in hip OA patients, using knee OA and non-OA control as comparator groups to identify if differences exist between hip and knee OA. Furthermore in the hip OA cohort, it was attempted to determine if there were any cytokine (s) that correlated with pain symptoms, as well as other clinical measurements.

## Methods

### Subjects and clinical evaluation

This study protocol was approved by the University of Calgary Human Research Ethics Board (REB15–0880). Participants (Table 1) provided written consent. In all patient cohorts a previous diagnosis of metabolic disease/disorder, diabetes and/or abdominal obesity excluded the individual from the current study. For all cohorts, prescription anti-inflammatory medication use within the past 3 months also excluded the individual from the current study. Individuals from all cohorts presented in this study are a sub-set of a larger on-going cohort study at the University of Calgary and were selected based on the inclusion/exclusion criteria for this study. Sample sizes were based off our previous reported study [5].

**Table 1:** Demographics of 3 cohorts

Characteristic	Knee OA (*n* = 100)	Hip OA (*n* = 50)	Control (*n* = 100)
Gender (% female)	46	42	75
Age (SD)	60.2 (10.4)	59.0 (9.5)	40.0 (9.5)
K/L 0	0	0	100
K/L 1	72	0	0
K/L 2	1	0	0
K/L 3	28	14	0
K/L 4	0	36	0
Serum Sample	100	50	100
Synovial Fluid Sample	8	14	15^a^
Synovium Sample	5	5	5^a^

^a^these sample are collected from cadaveric donations

#### Control (*n* = 100; mean age 40.0 ± 9.5 years)

Individuals showed no clinical signs of OA or RA (based on ACR criteria), and were questioned (and potentially excluded) regarding personal (intraarticular joint injury, inflammatory arthritis, autoimmune diseases) and family (inflammatory arthritis including any autoimmune diseases) histories.. Individuals (e.g. faculty, staff, students, volunteers) were recruited from the sports medicine program and human performance laboratory.

#### Knee osteoarthritis (*n* = 100; mean age 60.4 ± 10 years)

Inclusion criteria required a diagnosis of OA performed at the OA clinic (University of Calgary) based on clinical symptoms of 3 months or greater with radiographic (standing AP radiographs) evidence (Kellgren and Lawerence (K/L) grading: all radiographs were graded by a sports medicine physician and registered nurse first assistant: inter-reader reliability/kappa, κ = 0.84) of changes associated with OA in accordance with ACR criteria. A K/L grade of at least 1 was required for inclusion into the knee OA cohort. A previous diagnosis of hip, hand or spine OA resulted in exclusion and a clinical assessment was undertaken to identify potential undiagnosed OA in these joint was undertaken at the time of recruitment.

#### Hip osteoarthritis (*n* = 50; mean age 59 ± 9.5 years)

Inclusion criteria was based on a diagnosis of hip OA determined by patient symptoms, clinical exam and radiographic (K/L) evidence (all radiographs were graded by an orthopedic surgeon and registered nurse first assistant: κ = 0.87) in accordance with ACR criteria. A K/L grade of at least 1 was required for inclusion into the hip OA cohort. A previous diagnosis of knee, hand or spine OA resulted in exclusion and a clinical assessment was undertaken to identify potential undiagnosed OA in these joint was undertaken at the time of recruitment. Two major etiologies were impingement (*n* = 35) and dysplasia (*n* = 7).

### Clinical assessment of hip OA cohort

Patients were asked to consent to complete the following questionnaires at the time of surgery (Birmingham hip resurfacing or total hip replacement):

1. The Harris Hip score (HHS) [31]. The score has a maximum of 100 points (best possible outcome) covering pain (1 item, 0–44 points), function (7 items, 0–47 points), absence of deformity (1 item, 4 points), and range of motion (2 items, 5 points).
2. Hip disability and osteoarthritis outcome score (HOOS) [32]. It consists of 40 items assessing 5 subscales including pain, symptoms, activity limitations daily living, function in sport and recreation and hip related quality of life. Standardized answer options are given in 5 Likert-boxes with scores from 0 to 4 (no, mild, moderate, severe and extreme).
3. Short form 36 (SF36) [33]. It consists 8 sections including vitality, physical functioning, bodily pain, general health perceptions, physical role functioning, emotional role functioning, social role functioning, mental health. Each measurement is scale from 0 (worst) to 100 (best).
4. The University of California Los Angeles (UCLA) activity score [34]. The evaluation has 10 descriptive activity levels ranging from wholly inactive and dependent on others (level 1), to regular participation in impact sports (level 10).

The questionnaires were the most common ones in the literature concerning hip function and symptoms, and all of them had shown a good reliability.

### Sample collection

Serum samples were collected at rest by standard venipuncture with untreated vacuum tubes. Serum was immediately aliquoted and stored at -80 °C until required for analysis. All samples analyzed were only thawed once (at the time of analysis).

Synovial fluid (knee and hip OA cohorts) was aspirated from the knee joint by the attending orthopedic surgeon or sports medicine physician. Control synovial fluid samples were obtained from the Southern Alberta Tissue Donation Program. Criteria for control cadaveric donations were an age of 40 years or older, no history of arthritis, joint injury or surgery (including visual inspection of the cartilage surfaces during recovery), no prescription anti-inflammatory medications, no co-morbidities (such as diabetes/cancer), and availability within 4 h of death. Synovial fluid samples were collected without the use of lavage. The samples centrifuged at 3000 g for 15 min after blood has clotted at 4 °C and stored at -80 °C. All samples analyzed were only thawed once (at the time of analysis).

### Synovium tissue collection

Control tissue samples were obtained from the Southern Alberta Tissue Donation Program based on the sample criteria for cadaveric donations as listed above. Synovial biopsies from OA subjects were taken during surgery.

### Multiplexed arrays

Sample analysis was performed by Eve Technologies (Calgary, AB Canada) using the Milliplex MAP Human Cytokine/Chemokine Panel (Millipore), according to the manufacturer’s instructions. All samples (serum and synovial fluid) were assayed at least in duplicate and prepared standards were included in all runs. The following proteins were examined by Luminex in this study: EGF, Eotaxin, FGF2, Flt3L, Fractalkine, GCSF, GMCSF, GROα, IFNα2, IFNγ, IL1α, IL1β, IL1rα, IL2, IL3, IL4, IL5, IL6, IL7, IL8, IL9, IL10, IL12 (p40), IL12 (p70), IL13, IL15, IL17A, IL18, IP10, MCP1, MCP3, MDC, MIP1α, MIP1β, PDGFAA, PDGFAB/BB, RANTES, sCD40L, TGFα, TNFα, TNFβ, VEGFA. The sensitivities of these makers range from 0.1–10.1 pg/mL (average 2.359 pg/ml) and the inter-array accuracies range from 3.5% – 18.9% coefficient of variation (average 10.7%).

### Immunohistochemistry

Immunofluorescence was performed on synovium specimens with fluorescent-conjugated antibodies were obtained as follows: PE Mouse Anti-Human MDC/CCL22 (Cat. No. 565950, BD), PE Mouse Anti-Human IP-10 (Cat. No. 555049, BD), FITC Rat Anti-Human IL-6 (Cat. No. 554544, BD).

### Statistical methods

The normality of each cytokines was assessed by QQ plots. Because many of the cytokine concentrations were not normally distributed, both t-test and Mann–Whitney–Wilcoxon (MWW) tests were utilized. T-test and MWW were performed to compare the ages and BMI between groups. Principle component analysis (PCA) was used to reduce the dimensionality of the cytokine dataset and the first 3 components were used for 3-D scatter plot.

For hip OA cohort, T-test and MWW were used to compare the means of cytokines with pain scores, BMI between two subgroups-impingement and dysplasia. PCA was run to extract two factors from 10 HOOS pain questions. Pairwise associations between cytokines and clinical questionnaire scores were assessed using Spearman’s rank correlation test. First, we tested the correlation between 7 cytokines which have been reported correlated with osteoarthritis pain previously [27] and pain levels (evaluated by HOOS pain sub scores and SF36 body pain score). Then, we screened all the associations between every single cytokines and the log transformed ratios of any two cytokines and clinical variables (i.e., age, gender, BMI and clinical questionnaire scoring). *P*-values were adjusted by two different multiple testing correction procedures: Bonferroni procedure with familywise error rate of 0.05 (α ≈ 0.001) and Benjamini–Hochberg procedure with a false discovery rate of 0.20.

For both hip and knee OA cohorts, multivariable linear regression was applied to test the potential confounding effects including age, gender, K/L grade and affected joint (hip or knee) on cytokine profiles. Statistical analyses were performed using Python version 2.7 with scipy package (for screening associations between all cytokines and clinical questionnaire scores) and SPSS 20.0 (SPSS, Inc., Chicago IL). *P* < 0.05 (two-sided) was considered statistically significant, except for the tests whose *p*-values were adjusted by multiple testing correction. The 3-D scatter plots of PCA were generated by SPSS 20.0. The bar graphs were generated by GraphPad Prism 6 (Graphpad Software. San Diego, CA).

## Results

### Inflammatory profile between hip and knee OA cohorts

**Fig. 1:**
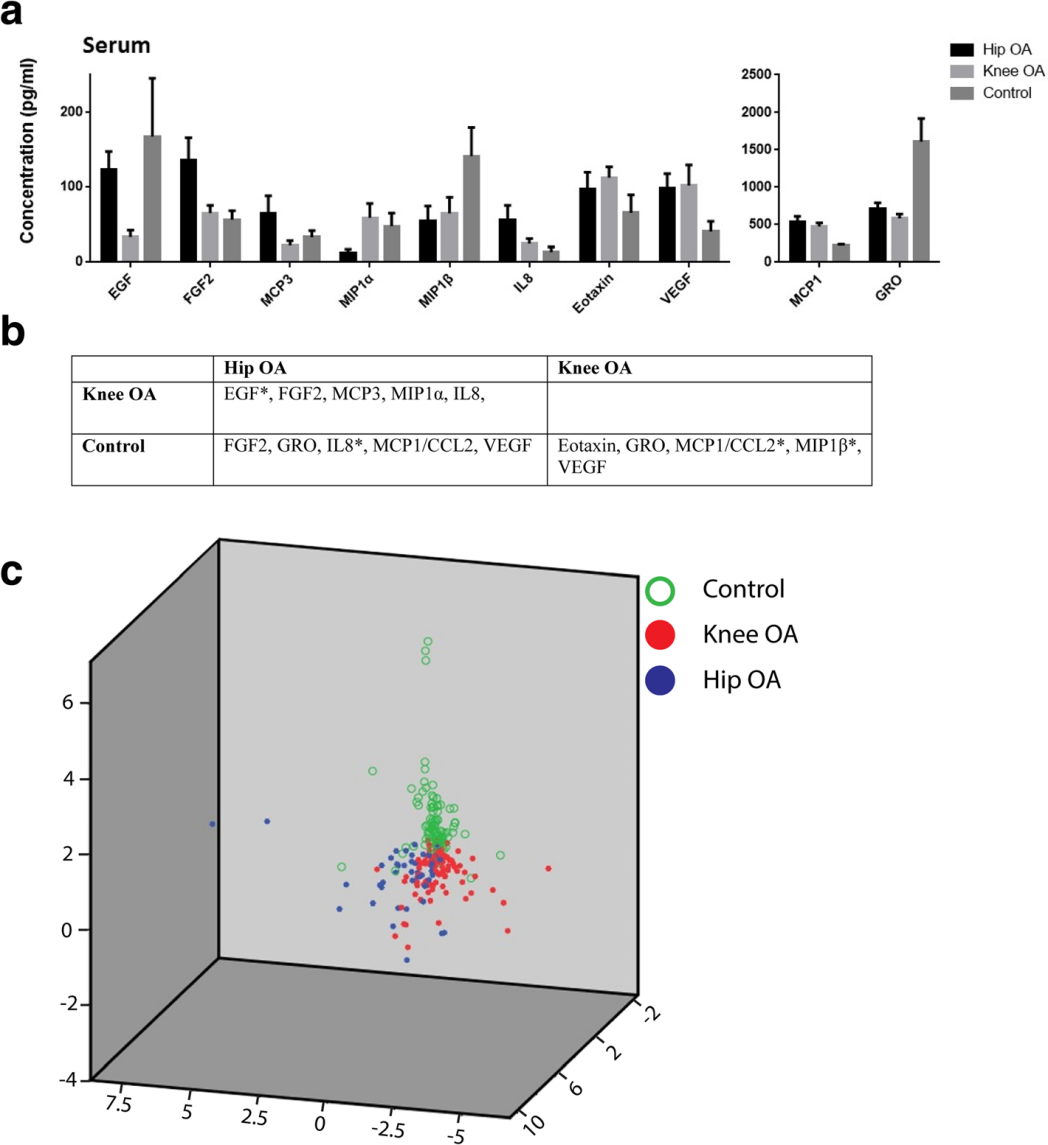
Comparison of cytokine profiles in serum in control, hip OA and knee OA cohorts. The serum concentrations of cytokines that were found to be significantly differently between the cohorts examined (**a**). The significantly different cytokines in each cohort are present in more detail in (**b**) after Bonferroni correction (*p* < 0.0012) (* = Significantly different in both serum and synovial fluid). Scatter plot of first 3 components of PCA of cytokine profiles of 3 cohorts demonstrates that the serum cytokine profiles are capable of discriminating the 3 different cohorts (**c**)

To uncover any differences in systemic inflammatory signaling in patients with hip OA vs knee OA or normal controls a multiplex approach was utilized in where we would quantify the levels of 42 cytokines (including lymphokines, interferons, colony stimulating factors and chemokines, outlined in methods section) that are involved in many signaling pathways regulating inflammation and disease. Significant differences in serum cytokines levels were identified when comparing cohorts (hip OA vs. knee OA vs. control). While 5 cytokines were differentially expressed between knee and hip OA cohorts (Fig. 1), 3 cytokines (GRO: growth-regulated oncogene aka C-X-C motif ligand 1/CXCL1, MCP1: monocyte chemoattractant protein aka C-C motif ligand 2/CCL2 and VEGF: vascular endothelial growth factor) were conserved in both hip and knee OA cohorts when compared to the control cohort (Fig. 1). Overall, 10 cytokines were differentially expressed between all three cohorts. The first 3 components of PCA (explained 64.5% of total variance) clearly separated 3 cohorts (Fig. 1).

**Fig. 2:**
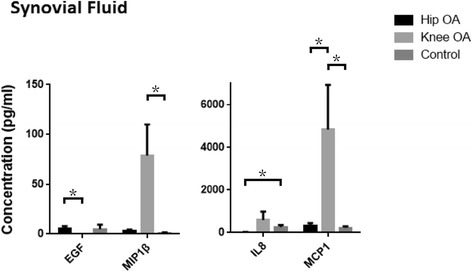
Differently expressed cytokines in synovial fluid in control, hip OA and knee OA cohorts. Of the cytokines examined in the serum that were significantly different between the hip OA, knee and control cohorts; EGF, MCP1, MIP1β and IL8 protein levels were also found to be significantly different between the synovial fluid samples obtained from the cohorts. Bonferroni correction (*p < 0.001)

Synovial fluid cytokine profiles were also found to be distinct across 3 cohorts. Synovial fluid and serum shared common cytokines that were differentially expressed between cohorts: EGF (epidermal growth factor) differed between hip OA and knee OA; IL8 (interleukin 8) was different between hip OA and control; MCP1, MIP1β (macrophage inflammatory protein aka C-C motif ligand 3/CCL3) differed between knee OA and control (Fig. 2).

Previous studies showed age, gender and severity of OA might have effects on cytokine expression [35, 36]. Therefore, a multivariable linear regression model was applied to access the correlations between cytokines and potential confounding variables (age, gender and K/L grade of OA and affected joint) in OA cohorts (Table 2). For cytokines that were significantly different between hip and knee OA cohorts, none were found to be related to age, gender or K/L grade, suggesting there were minimal effects of these variables confounding the cytokine profiles observed. After adjusting for confounding variables, EGF, FGF2 (fibroblast growth factor 2), MCP3 (aka C-C motif ligand 7/CCL7), and IL8 remained different between hip and knee OA.

**Table 2:** Multivariable linear regression result of associations between cytokines and age, gender, K/L grade and affected joint (e.g. knee vs. hip)

Cytokines	Covariates	Sig.	95% CI	
			Lower	Upper
EGF	Age	0.396	−1.440	0.573
	Gender	0.177	−34.025	6.343
	K/L Grade	0.437	−7.964	18.331
	Affected Joint*	< 0.001	−37.895	−14.098
FGF2	Age	0.370	−1.761	0.659
	Gender	0.195	−40.270	8.269
	K/L Grade	0.803	−13.809	17.809
	Affected Joint *	0.003	−36.048	−7.434
MCP3	Age	0.778	−0.751	1.001
	Gender	0.238	−28.085	7.046
	K/L Grade	0.909	−12.108	10.776
	Affected Joint *	0.005	−25.219	−4.508
MIP1α	Age	0.863	−1.426	1.197
	Gender	0.298	−40.207	12.400
	K/L Grade	0.572	−22.039	12.228
	Affected Joint	0.130	−3.555	27.458
IL8	Age	0.799	−0.881	0.680
	Gender	0.544	−20.479	10.832
	K/L Grade	0.731	−8.422	11.973
	Affected Joint *	0.049	−18.501	−0.042

*p < 0.05

### Correlations between cytokines and clinical data in hip OA cohort

To determine if any clinical outcome measures were associated with the cytokine profile of hip OA patients, the pain, physical function and activity limitations of hip OA cohort were assessed using the HOOS, SF-36, HHS and UCLA scores.

First the discriminativity of all clinical measurements was examined by comparing each measurement between different severity of OA (different K/L grade). Six sub-scores showed a difference between K/L grade 3 and 4 groups (significant threshold was corrected by Benjamini–Hochberg procedure with a false discovery rate of 0.20) (Additional file 1: Table S2). These scores included the HOOS pain 5 (walking on a flat surface, *p*-value = 0.037), HOOS pain 6 (going up or down stairs, *p*-value = 0.034), SF36 GH (general-health, *p*-value = 0.023), SF36 MH (mental health, *p*-value = 0.007), SF36 PCS (physical component summary, *p*-value = 0.018) and SF36 MCS (mental component summary, *p*-value = 0.024). None were significant after Bonferroni testing correction.

**Fig. 3:**
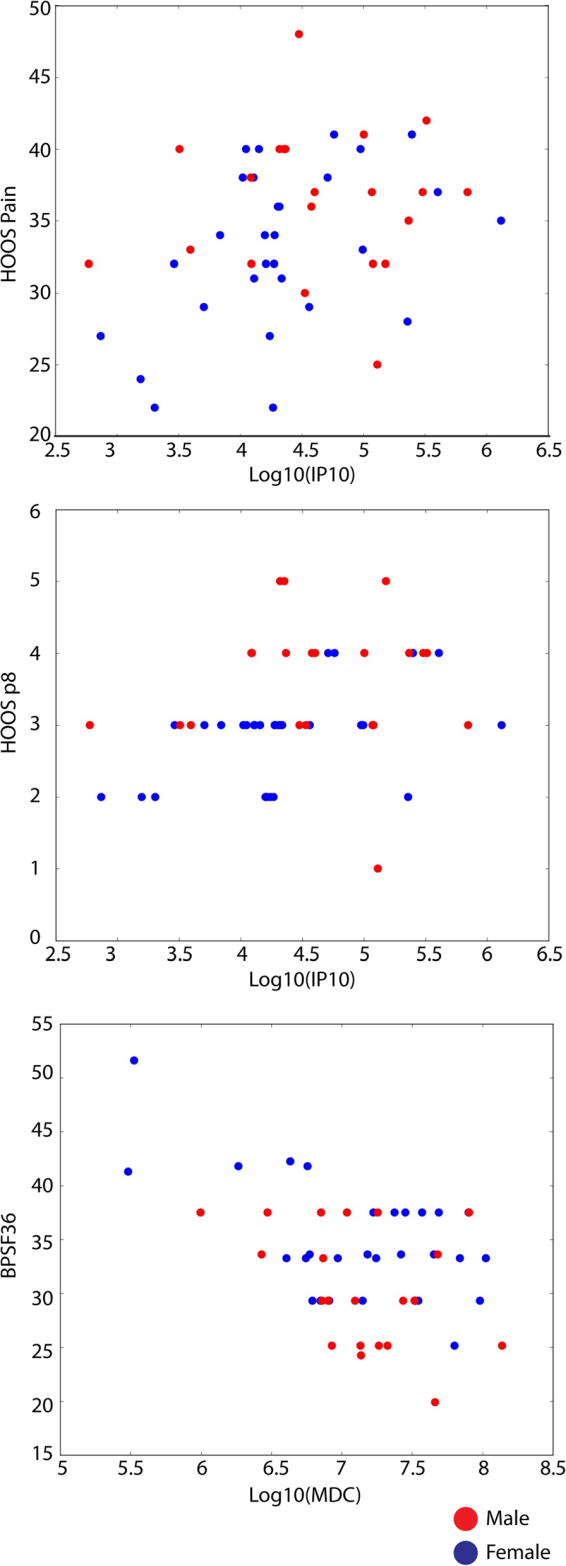
Scatter plot of two cytokines and correlated pain scores. Left: IP10 (log transferred) was positively correlated with HOOS pain score (correlation = 0.294, *p*-value = 0.038). Middle: IP10 (log transferred) was positively correlated with HOOS pain question 8 (correlation = 0.390, *p*-value = 0.005). Right: MDC (log transferred) was negatively correlated with SF36 body pain score (correlation = − 0.302, *p*-value = 0.033)

Previously reported OA pain related cytokines (TNFα, IL1β, IL6, IL15, IL10, MCP1 and Fractalkine) [27] were also examined within our hip OA cohort dataset (Additional file 1: Table S3). Only IL6 was found to be significant when correlated with HOOS pain score factor 2 (correlation = 0.319, *P*-value = 0.024). When testing all 36 cytokines, 2 cytokines were found to be correlated with pain severity: MDC (macrophage-derived chemokine aka C-C motif chemokine 22/CCL22) was negatively correlated with BPSF36 (body pain SF36, correlation = − 0.302, *p*-value = 0.033), IP10 was positively correlated with HOOS pain (correlation = 0.294, *p*-value = 0.038) and HOOS pain 8 (standing upright, correlation = 0.390, *p*-value = 0.005) (Fig. 3).

### Synovium examination from control, hip OA and knee OA cohorts

**Fig. 4:**
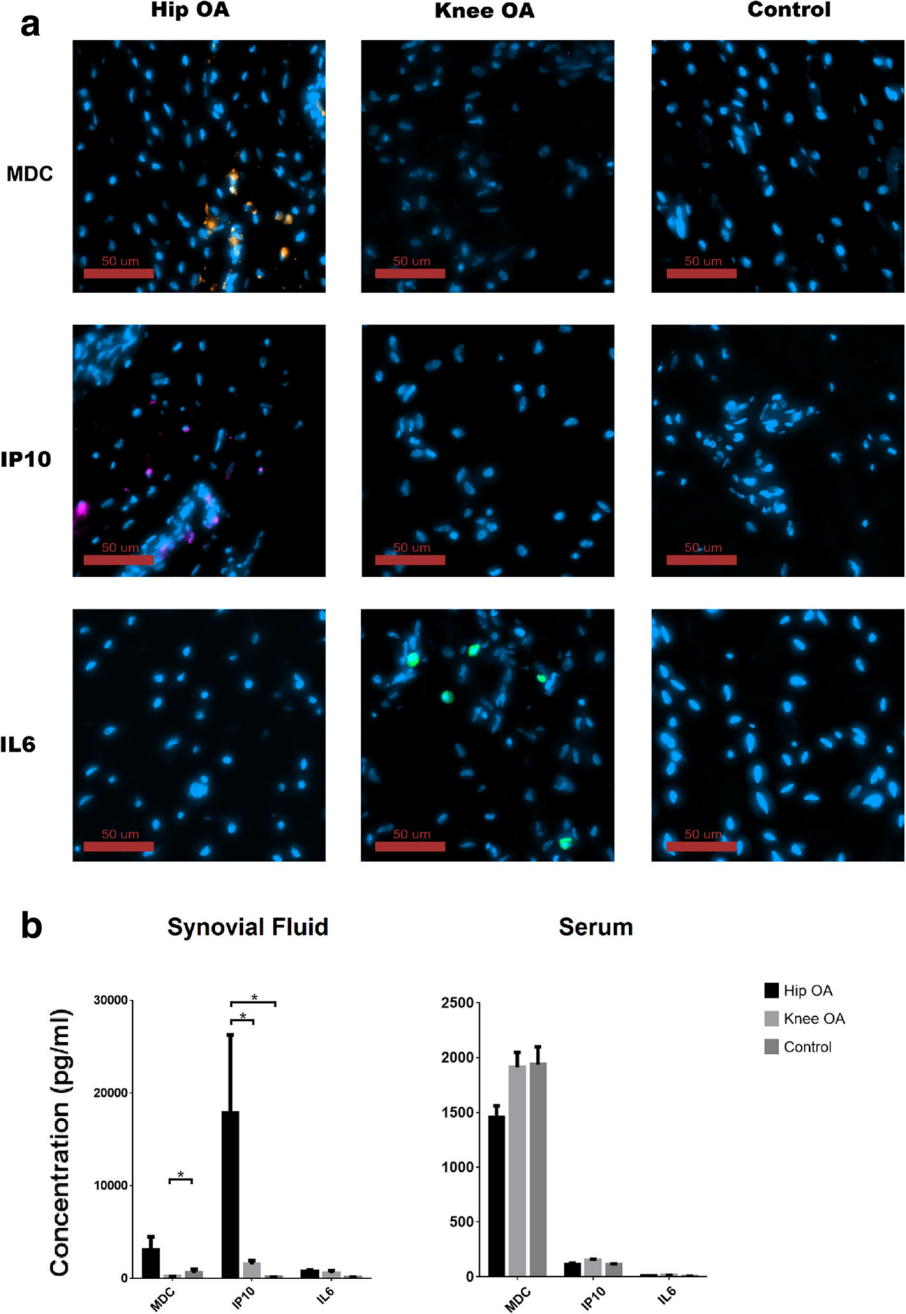
Presence of MDC, IL6 and IP10 in Hip OA, Knee OA and control synovium, synovial fluid and serum. Representative images demonstrating the presence of MDC (orange) positive (MDC: 3/5 hip OA, 0/5 knee OA, 0/5 control) and IP10 (purple) positive (3/5 hip OA, 0/5 knee OA, 0/5 control) cells in synovium of Hip OA patients. IL-6 (green) positive cells within the synovium were not observed in the Hip OA cohort, but observed with the knee OA cohort (1/5 hip OA, 4/5 knee OA, 0/5 control) (**a**). Serum and synovial fluid levels of MDC, IL6 and IP10 were also examined in the three cohorts (**b**). Bonferroni correction (*p < 0.001)

To determine if the cytokines that were correlated with hip pain could be originating from the joint environment, the expression of MDC, IL6 and IP10 were examined in synovial membrane biopsies from a sub-set of the hip OA (*n* = 5), knee OA (*n* = 5) and control cohorts (*n* = 5) (Fig. 4). MDC and IP10 positive cells were observed in the synovium of patients with hip OA. IL6 positive cells were not regularly observed in the synovium of hip OA patients, but were observed in the synovium of patients with knee OA. MDC, IL6 or IP10 positive cells were not observed within the synovium of patients without OA. To further narrow down the potential source of the MDC, IL6 and IP10, synovial fluid and serum samples were examined in a sub-set of patients. High levels of MDC were observed in the serum of all cohorts, however, only patients with hip OA maintained high levels of MDC within their synovial fluid (Fig. 4 and Additional file 1: Figure S1). While IP10 was present in serum at lower levels, high levels were observed in the synovial fluid of hip OA patients alone. IL6 levels were present at low levels in serum and synovial fluid of all cohorts examined (Fig. 4 and Additional file 1: Figure S1). It is important to note that although multiple comparisons were not undertaken for this experiment, we chose to present the data (Fig. 4) with the same multiple comparison correction as the previous results for consistency. However, a non-multiple comparison corrected version of the same data is presented in Additional file 1: Figure S1.

## Discussion

To our knowledge, few papers have discriminated which joint the OA is affecting when comparing OA with control groups. In this study, we have found that 4 cytokines (EGF, FGF2, MCP3, IL8) are expressed differently in knee OA patients versus hip OA patients after controlling for age, gender and K/L grade. Interestingly, when we compared these two OA cohorts with the control cohort, the picture became even more complex. EGF levels were similar between the hip OA and control cohorts but both differed from the knee OA cohort. MCP3 was similar between the knee OA and control cohorts but differed from the hip OA cohort. The scatter plot of first 3 components of PCA of the 3 cohorts (hip OA, knee OA and control) showed that, neither Hip OA nor Knee OA is “closer” to the control. The center of gravity of the 3 cohorts represents a triangle shape (Fig. 1). This infers that the three cohorts have their own characteristic cytokine profiles (though knee OA and hip OA cohorts are more similar to each other than either is to the control cohort). This may suggest that, at least for serum cytokines, OA may have distinct inflammatory profiles with subtypes that vary according to the affected joint. Failing to treat them as separate groups may be one of the reasons that the accuracy of biochemical markers in OA varies among studies [37]. This may partially explain why none of the biochemical markers that have been studied to date have been approved for diagnosis or prognosis of OA. Another possible reason might be the heterogenetic and multifactorial nature of OA itself. The existence of subgroups in OA might invalidate any statistical single biochemical marker comparison which considers OA as one group-one biochemical marker is not enough to classify all of these subgroups. Previous studies have looked into subgrouping OA before statistical analyses [38]. For instance, by using serum metabolomics data of OA patients, Zhang et al. identified at least 3 distinct subgroups which were not associated with any known confounders including age, sex, BMI and comorbidities [39]. The current study suggests that hip and knee OA cohorts may not be as similar in terms of the disease as previously thought and this has implications that OA in other joints (hands, spine, etc.) may also cloud diagnostic efforts, however, this hypothesis will require further study and validation.

In specific regards to the inflammatory cytokines examined in the current study, it is believed that aging brings about changes in the expression of many cytokines. Aged-related changes in the immune system may elevate the concentrations of cytokines which some have postulated may ultimately lead to chronic inflammation [40]. This being said, in our dataset none of the cytokines are statistically significantly correlated with age. This might be due to the fact that most patients have passed their middle-age and the age distribution of these two cohorts are relatively concentrated (mean = 59.9, SD = 10.1). Although the control cohort is significant younger than the two OA cohorts (mean = 40.0, SD = 9.5). A limitation of the current study is that we unfortunately do not have the linked age information for each patient sample in the control cohort (only the cohort level details) and therefore we are not able to run a correlation analysis. Additionally, many factors can confound cytokine concentrations, such as statistical errors and mismatched cohort characteristics (such as races, disease history, diet and etc.). While, it can be unrealistic to find two exactly matched cohorts, these mismatch factors may lead to changes in serum cytokine concentrations in an unpredictable way and highly controlled cohort studies will be required to address these discrepancies.

TNFα, IL1, IL6 and IL17 have been reported to be correlated with development of neuropathic pain in various animal models [27, 41]; however, except for IL6, which had weak correlation with PCA factor 2 of 10 HOOS pain questions, none of these proteins were significantly correlated with pain level scores in our dataset. We have tested the correlation between our two different pain level scores-SF36 body pain and HOOS pain sub-score, they are significantly correlated (correlation = 0.569, *p*-value < 0.0001). This suggests the method that we chose to evaluate osteoarthritis pain levels was reliable between questionnaires. Among all 36 cytokines, we found that MDC and IP10 were correlated with pain in the hip OA cohort. IP10 (CXCL10) belongs to CXC chemokine family. It is expressed by a variety of cell types. When IP10 binds to CXCR3, this can result in activation and recruitment of leukocytes, and also regulate cell growth and apoptosis [42]. In specific regard to pain, IP10 has been reported to be involved in breast cancer-induced bone pain by activation of microglia in rat models [43]. In our study, IP10 was positively correlated with pain in patients with hip OA. It is possible that IP10 could be inducing OA pain through a similar pathway by activation of microglia in the joint environment. MDC (CCL22) belongs to the CC chemokine family and it is expressed mainly by macrophages and dendritic cells. MDC recruits Th2, Th17 and regulatory T cells through the binding of trafficking receptor CCR4 [44, 45]. To our knowledge, no publication has directly looked at MDC and pain.

The concentrations of these cytokines in synovial fluid were consistent with the immuno-histochemistry results; with MDC and IP10 higher in synovial fluid than in serum, and were higher in hip OA than knee OA or control cohorts. This strongly suggests that within the hip OA cohort, MDC and IP10 are being produced within the joint environment and then may diffuse into serum through the synovial fluid. In the knee OA cohort however, MDC and IP10 were not present within the joint (synovium or synovial fluid) and therefore any detectable levels of these in the serum are most likely being generated in other tissues in the body. While, the concentration of IL6 was higher in hip and knee OA cohorts than controls, IL6 was not found in hip synovium. This suggests that there might be other tissues in the joint (cartilage, ligaments, etc) that produce IL6 in the hip OA cohort. The relationship between serum and synovial fluid cytokine levels are still poorly understood with previous studies demonstrating little correlation [46] or high correlation [47] between systemic vs. local levels. However, the biological basis behind these correlations (or lack thereof) remain unknown, and further study will be required on specific markers to determine if the choice of sample local will impact the usefulness of the result obtained.

## Conclusions

These findings validate previous studies that cytokines are differently expressed in OA patients [15]; and suggest that while cytokine profiles are generally similar between hip and knee OA, that there are specific differences that may be related to differential disease processes within a given joint. Furthermore, 3 cytokines (IL6, MDC and IP10) were identified that correlated with hip OA. These results set the ground work for future studies that will be required to understand: if cytokines play distinct roles in in the onset/pathogenesis of OA in different joints; and what is the pathway and/or mechanism by which the identified cytokines regulate OA pain.

## Additional file

Additional file 1: Figure S1. Presence of MDC, IL6 and IP10 in Hip OA, Knee OA and control synovium, synovial fluid and serum not corrected for multiple comparisons. **Table S1.** Biochemical markers that have been reported to be correlated with OA pain. **Table S2.** Questionnaire scores of hip OA cohort. **Table S3.** Correlations between cytokine concentrations and hip pain. (DOCX 140 kb)

## Abbreviations

ACR: American College of Rheumatology
BMI: Body mass index
COMP: Cartilage oligomeric matrix protein
EGF: Epidermal growth factor
FGF2: Fibroblast growth factor 2
GRO: Growth-regulated protein
HHS: Harris Hip score
HOOS: Hip disability and osteoarthritis outcome score
IL: Interleukin
IP: Interferon gamma-induced protein
K/L: Kellgren and Lawerence
MCP: Monocyte chemoattractant protein
MDC: Macrophage-derived chemokine
MIP: Macrophage inflammatory protein
MWW: Mann–Whitney–Wilcoxon test
OA: Osteoarthritis
PCA: Principle component analysis
SF36: Short Form 36
Th: T Helper
TNF: Tumor necrosis factor
UCLA: University of California Los Angeles activity score
VEGF: Vascular endothelial growth factor
