# Supplementary material for: Serum and synovial fluid cytokine profiling in hip osteoarthritis: distinct from knee osteoarthritis and correlated with pain
Source: BMC Musculoskelet Disord. 2018 Feb 5;19:39. doi: 10.1186/s12891-018-1955-4 (PMC5800026; doi:10.1186/s12891-018-1955-4)
Supplement: Additional file 1: Figure S1. — Presence of MDC, IL6 and IP10 in Hip OA, Knee OA and control synovium, synovial fluid and serum not corrected for multiple comparisons. Table S1. Biochemical markers that have been reported to be correlated with OA pain. Table S2. Questionnaire scores of hip OA cohort. Table S3. Correlations between cytokine concentrations and hip pain. (DOCX 140 kb) [file 12891_2018_1955_MOESM1_ESM.docx]

**
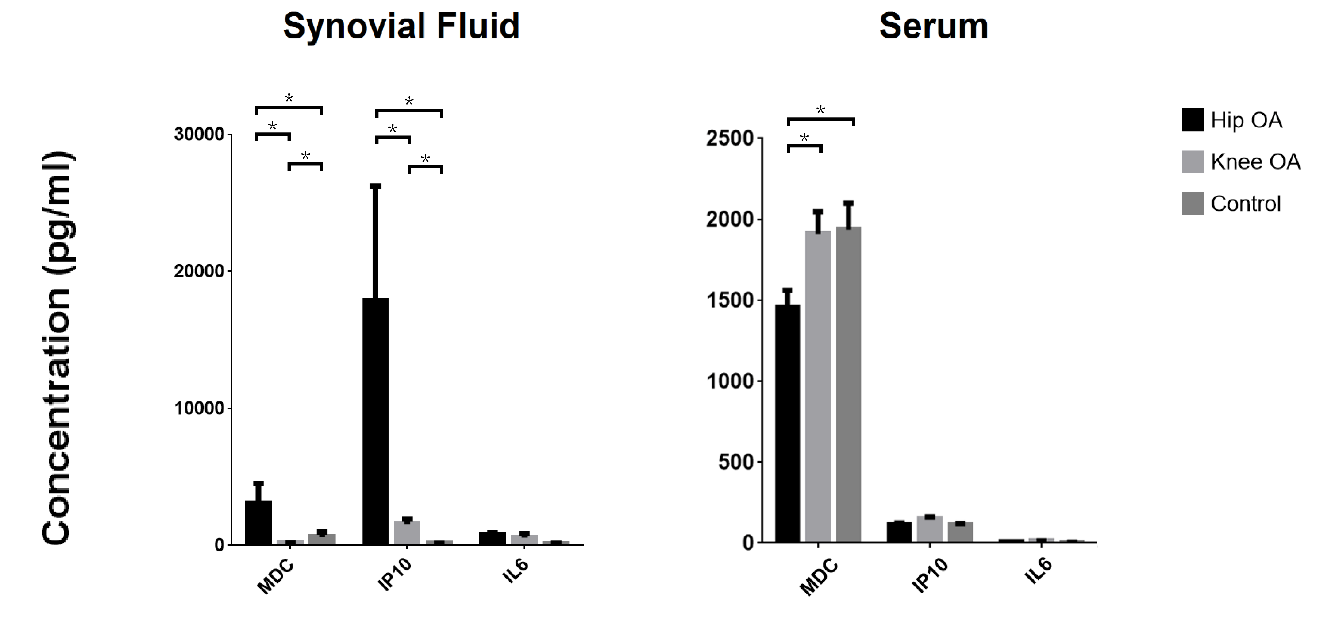
**

**Figure S1. Presence of MDC, IL6 and IP10 in Hip OA, Knee OA and** control **synovium, synovial fluid and serum not corrected for multiple comparisons.** Serum and synovial fluid levels of MDC, IL6 and IP10 were examined in the three cohorts. * p<0.05. Abbreviations used in figure legend: MDC (macrophage derived chemokine), IL6 (interleukin 6), IP10 (interferon gamma-induced protein 10), OA (osteoarthritis).

**Table S1. Biochemical markers that have been reported to be correlated with OA pain**

| **Marker** | **Pain Criterion** | **Population** | **OA Joint** | **Bodily Fluid** | **Sample Size** | **Citation #** |
| --- | --- | --- | --- | --- | --- | --- |
| CD163 | NHANES-I |  | Knee | Synovial fluid, blood | 184 | ([40](#_ENREF_40)) |
| CD14 | NHANES-I |  | Knee | Synovial fluid, blood | 184 | ([40](#_ENREF_40)) |
| NPY | Hideo Watanabe’s knee scoring system-related pain score |  | Knee | Synovial fluid | 100 | ([41](#_ENREF_41)) |
| COMP | AUSCAN pain |  | Hand | Serum | 663 | ([42](#_ENREF_42), [43](#_ENREF_43)) |
| IL15 | WOMAC pain score | Chinese | Knee | Serum | 226 | ([44](#_ENREF_44)) |
| IL6 | VAS, JKOM-pain | Japanese | Knee | Serum | 160 | ([45](#_ENREF_45)) |
| leptin | WOMAC and VAS |  | Knee, hip | Synovial fluid | 219 | ([46](#_ENREF_46)) |
| hs-CRP | WOMAC |  | Knee | Serum | 149 | ([47](#_ENREF_47)) |
| TNFα | WOMAC |  | Knee | Serum | 149 | ([47](#_ENREF_47)) |
| ARGS | KOOS pain |  | Knee | Synovial fluid | 141 | ([48](#_ENREF_48)) |
| CTXII | VAS | Japanese | Knee | Urine | 47 | ([49](#_ENREF_49)) |
| NTX | VAS | Japanese | Knee | Urine | 47 | ([49](#_ENREF_49)) |
| HA | VAS | Japanese | Knee | Serum | 47 | ([49](#_ENREF_49)) |
| CPII | VAS | Japanese | Knee | Serum | 47 | ([49](#_ENREF_49)) |

Abbreviations used in table: CD (cluster of differentiation), NPY (neuropeptide Y), COMP (cartilage oligomeric matrix protein), IL (interleukin), hs-CRP (high-sensitivity C-reactive protein), TNFα (tumor necrosis factor alpha), ARGS (aggrecan neo-epitope fragment), CTXII (carboxy-terminal telepeptides of type II collagen), NTX (n-telopeptides of type 1 collagen), HA (hyaluronan), CPII (proCollagen II c-propeptide), NHANES (National Health and Nutrition Examination Survey), AUSCAN (AUStralian CANadian Osteoarthritis Hand Index), WOMAC (Western Ontario and McMaster Universities Osteoarthritis Index), VAS (Visual Analogue Scale), JKOM (Japanese Knee Osteoarthritis Measure), KOOS (Knee injury and Osteoarthritis Outcome Score).

**Table S2. Questionnaire scores of hip OA cohort.**

| **Questionnaire scores** | **K/L grade 3**  **(mean ± SD)** | **K/L grade 4**  **(mean ± SD)** | **P-value** | **Benjamini-Hochberg** **critical value** |
| --- | --- | --- | --- | --- |
| MHSF36 | 44.6 ± 8.1 | 52.3 ± 9.7 | 0.007** | 0.007407 |
| GHSF36 | 48.8 ± 6.8 | 54.2 ± 7.8 | 0.013* | 0.014815 |
| PCSSF36 | 32.8 ± 8.2 | 27.0 ± 5.7 | 0.018* | 0.022222 |
| MCSSF36 | 45.9 ± 10.8 | 54.9 ± 11.8 | 0.024* | 0.02963 |
| HOOSp6 | 2.5 ± 0.9 | 3.1 ± 0.9 | 0.034* | 0.037037 |
| HOOSp5 | 3.1 ± 0.7 | 3.7 ± 0.9 | 0.037* | 0.044444 |
| PFSF36 | 31.4 ± 9.4 | 25.5 ± 5.6 | 0.052 | 0.051852 |
| HOOS | 48.4 ± 12.9 | 41.1 ± 11.0 | 0.082 | 0.059259 |
| HOOS Pain | 32.1 ± 6.25 | 35.4 ± 5.0 | 0.083 | 0.066667 |
| HOOS Pain factor 1 | 0.41 ± 1.14 | 0.16 ± 0.91 | 0.084 | 0.074074 |
| HOOSp1 | 4.1 ± 0.7 | 4.5 ± 0.5 | 0.104 | 0.081481 |
| HOOSp10 | 3.5 ± 0.6 | 3.8 ± 0.8 | 0.176 | 0.088889 |
| HOOSp9 | 3.2 ± 0.8 | 3.5 ± 0.7 | 0.178 | 0.096296 |
| BPSF36 | 34.3 ± 4.9 | 32.5 ± 6.2 | 0.184 | 0.103704 |
| RPSF36 | 36.0 ± 10.7 | 32.1 ± 7.6 | 0.195 | 0.111111 |
| RESF36 | 38.8 ± 14.7 | 43.6 ± 14.4 | 0.199 | 0.118519 |
| HOOSp2 | 3.1 ± 0.9 | 3.4 ± 0.7 | 0.21 | 0.125926 |
| HOOSp4 | 3.0 ± 0.8 | 3.3 ± 0.8 | 0.224 | 0.133333 |
| SFSF36 | 37.1 ± 8.6 | 39.8 ± 11.8 | 0.33 | 0.140741 |
| HOOSp8 | 3.0 ± 0.8 | 3.2± 0.9 | 0.362 | 0.148148 |
| HOOSp7 | 3.0 ± 0.8 | 3.1 ± 0.8 | 0.432 | 0.155556 |
| UCLA | 4.4 ± 1.8 | 4.0 ± 1.6 | 0.458 | 0.162963 |
| HHS | 53.0 ± 11.9 | 56.6 ± 12.1 | 0.596 | 0.17037 |
| HOOSp3 | 3.6 ± 0.6 | 3.6 ± 0.8 | 0.68 | 0.177778 |
| BMI | 32.4 ± 4.0 | 35.4 ± 5.0 | 0.871 | 0.185185 |
| VTSF36 | 44.8 ± 9.6 | 46.2 ± 9.6 | 0.871 | 0.192593 |
| HOOS Pain factor 2 | 0.02 ± 0.66 | 0.01 ± 1.11 | 0.931 | 0.2 |

|  | | **HOOS** | | | | | | | | | | | | | **BPSF36** |
| --- | --- | --- | --- | --- | --- | --- | --- | --- | --- | --- | --- | --- | --- | --- | --- |
|  |  | **Total** | **1** | **2** | **3** | **4** | **5** | **6** | **7** | **8** | **9** | **10** | **PC1** | **PC2** |  |
| **Fractalkine** | **Corr.** | 0.18 | -0.063 | -0.001 | -0.217 | -0.268 | -0.096 | -0.095 | -0.032 | -0.089 | -0.158 | -0.107 | -0.19 | 0.089 | -0.067 |
|  | **P-value** | 0.212 | 0.664 | 0.994 | 0.131 | 0.06 | 0.506 | 0.51 | 0.825 | 0.54 | 0.275 | 0.461 | 0.185 | 0.54 | 0.646 |
| **IL10** | **Corr.** | 0.091 | -0.047 | -0.183 | -0.193 | -0.016 | 0.061 | 0.021 | -0.22 | -0.124 | 0.044 | 0.01 | -0.085 | -0.045 | -0.099 |
|  | **P-value** | 0.53 | 0.746 | 0.202 | 0.18 | 0.912 | 0.673 | 0.884 | 0.125 | 0.392 | 0.763 | 0.944 | 0.558 | 0.756 | 0.493 |
| **IL15** | **Corr.** | 0.009 | -0.101 | 0.077 | -0.102 | -0.029 | 0.126 | 0.031 | -0.073 | -0.096 | 0.043 | 0.13 | 0.007 | -0.019 | -0.179 |
|  | **P-value** | 0.953 | 0.484 | 0.595 | 0.483 | 0.841 | 0.384 | 0.832 | 0.615 | 0.506 | 0.765 | 0.37 | 0.96 | 0.896 | 0.214 |
| **IL6** | **Corr.** | 0.036 | 0.285* | 0.061 | -0.014 | -0.031 | 0.201 | -0.081 | -0.178 | -0.213 | 0.05 | 0.025 | -0.006 | -0.319* | -0.057 |
|  | **P-value** | 0.804 | 0.045* | 0.676 | 0.921 | 0.831 | 0.161 | 0.574 | 0.217 | 0.138 | 0.728 | 0.865 | 0.966 | 0.024* | 0.696 |
| **MCP1** | **Corr.** | 0.028 | -0.128 | -0.078 | 0.042 | -0.044 | 0.084 | -0.025 | -0.04 | -0.046 | -0.045 | -0.016 | -0.044 | 0.006 | 0.086 |
|  | **P-value** | 0.848 | 0.374 | 0.589 | 0.771 | 0.762 | 0.564 | 0.862 | 0.782 | 0.75 | 0.754 | 0.911 | 0.763 | 0.967 | 0.553 |
| **TNFa** | **Corr** | -0.039 | 0.083 | 0.202 | 0.003 | -0.019 | 0.169 | -0.071 | -0.057 | -0.008 | 0.137 | 0.022 | 0.033 | -0.131 | -0.146 |
|  | **P-value** | 0.79 | 0.568 | 0.159 | 0.986 | 0.895 | 0.242 | 0.623 | 0.697 | 0.953 | 0.342 | 0.88 | 0.82 | 0.363 | 0.312 |
| **IP10** | **Corr.** | -.294* | 0.11 | 0.198 | 0.218 | 0.089 | 0.122 | 0.167 | 0.233 | .390** | 0.231 | 0.142 | -0.223 | 0.11 | 0.198 |
|  | **P-value** | 0.038* | 0.447 | 0.168 | 0.128 | 0.54 | 0.397 | 0.246 | 0.103 | .005** | 0.106 | 0.326 | 0.12 | 0.447 | 0.168 |
| **MDC** | **Corr.** | 0.048 | 0.018 | 0.085 | 0.085 | -0.077 | 0.109 | 0.03 | -0.041 | -0.071 | 0.005 | 0.064 | -.302* | -0.048 | 0.018 |
|  | **P-value** | 0.74 | 0.901 | 0.558 | 0.558 | 0.594 | 0.45 | 0.838 | 0.779 | 0.626 | 0.971 | 0.659 | .033* | 0.74 | 0.901 |

**Table S3. Correlations between cytokine concentrations and hip pain**

Abbreviations used in table: IL (interleukin), MCP (monocyte chemoattractant protein), MDC (macrophage derived chemokine), IP10 (interferon gamma-induced protein), TNF (tumor necrosis factor), PC (principle component), HOOS (Hip disability and osteoarthritis outcome score), SF36 (Short Form 36).
